# Preschool depression: clinical course, risk pathways, and early intervention strategies

**DOI:** 10.3389/fpsyt.2025.1685131

**Published:** 2025-11-26

**Authors:** Maria Pontillo, Cristina Di Vincenzo, Ilaria Bertoncini, Chiara Pastore, Valeria Villani, Giulia D’Amario, Stefano Vicari

**Affiliations:** 1 Child and Adolescent Neuropsychiatry Unit, Bambino Gesù Children’s Hospital, IRCCS, Rome, Italy; 2 Deparment of Psychology, Catholic University of the Sacred Heart, Milan, Italy; 3 Department of Neuroscience, Catholic University of the Sacred Heart, Rome, Italy; 4 Department of Life Science and Public Health, Catholic University of the Sacred Heart, Rome, Italy

**Keywords:** preschool depression, risk factor, trajectories, treatment, early intervention

## Abstract

Preschool-onset depression is increasingly recognized as a valid and clinically significant disorder, distinct from developmentally normative sadness and irritability. This mini-review synthesizes principal findings on its clinical presentation, risk and protective factors, developmental course, and evidence-based treatments. A narrative review approach was adopted. Research studies were selected based on key topics, including preschool depression, associated risk and protective factors, developmental trajectories, and treatment approaches, using PubMed as the primary database. Studies were selected based on their relevance to preschool-aged children (3-6 years) and their contribution to the understanding of clinical presentation, longitudinal course, etiological mechanisms, and intervention strategies. Included sources comprised longitudinal cohort studies, randomized controlled trials, and neuroimaging investigations. Preschool depression is associated with specific symptom patterns, such as anhedonia, guilt, and emotional dysregulation. Key risk factors include genetic predisposition, parental psychopathology, early adversity, and temperamental vulnerability. Protective factors such as effortful control and secure attachment appear to mitigate developmental risk. Longitudinal studies confirm both homotypic and heterotypic continuity into adolescence. Among emerging treatments, Parent-Child Interaction Therapy – Emotion Development shows strong empirical support, targeting both symptom reduction and emotional competence. Preschool depression represents a distinct condition with significant implications for long-term mental health. Early identification and intervention, particularly those enhancing regulatory capacities and caregiver-child relationships, are critical to altering maladaptive trajectories and improving outcomes.

## Introduction

Historically, depressive symptoms in preschool-aged children were often dismissed as transient or developmentally normative. However, foundational work by Kashani and colleagues challenged this view by identifying depressive symptoms in children aged 2,5 to 6 years that, while not fully meeting DSM criteria, were nonetheless clinically concerning. These findings prompted a re-evaluation of existing diagnostic frameworks and highlighted the need for developmentally sensitive diagnostic criteria for early-onset depression (1, 2).

Therefore, Luby and colleagues introduced and validated age-appropriate criteria for preschool-onset major depressive disorder, an impairing clinical condition, characterized by distinct symptom constellations and functional impairments across emotional, social, and cognitive domains when compared to healthy control (3–5). Notably, preschool depression is associated with physiological markers such as heightened cortisol reactivity to stress, a feature commonly observed in adult depressive disorders (6). Longitudinal studies have also provided strong evidence for the content and discriminant validity of the diagnosis, as well as its homotypic continuity with major depressive disorder in later childhood and adolescence (5, 7, 8). Building on these early findings, subsequent research has further refined the clinical profile of preschool depression, highlighting its stability, familial aggregation, and distinct neurodevelopmental features (1, 4, 5, 7, 9).Neuroimaging studies, for instance, have identified functional brain changes analogous to those seen in depressed adults in both school-age children with a history of preschool depression and currently depressed preschoolers (10–12).

Recent studies have further clarified the frequency and severity thresholds that distinguish clinically significant depressive symptoms in early childhood. Bufferd et al. (13) differentiated between developmentally normative symptoms, such as sadness or irritability, and non-normative markers like anhedonia, low self-worth, and suicidal ideation. For instance, low self-worth occurring just twice over two weeks was considered clinically significant, whereas sadness required a much higher frequency to meet threshold (13). These findings highlight the importance of contextualizing symptom expression within developmental norms and reinforce earlier observations of symptom specificity, such as the high specificity of guilt to preschool depression (5).

Epidemiological studies across diverse populations have converged on a prevalence rate of approximately 2% for preschool depression (14, 15). Recent comprehensive reviews also affirmed that preschool depression is a validated diagnostic entity affecting 2% of 3–5 years old, with identifiable clinical, neurobiological, and developmental correlates (16). In addition, several investigations consistently report high rates of comorbidity with anxiety disorders, attention-deficit/hyperactivity disorder (ADHD), oppositional defiant disorder (ODD), and conduct disorder (CD), further underscoring the clinical relevance of depression in this age group (15, 17).

Critically, longitudinal studies have confirmed the developmental continuity of preschool depression into later childhood and adolescence. For example, Finsaas et al. (17) demonstrated homotypic continuity of depressive symptoms from ages 4 to 10, attributing this pattern to stable familial factors such as genetic predisposition and consistent parenting styles (17). Similarly, other studies have shown that children with preschool depression are at significantly higher risk of developing full-threshold MDD after puberty (18). These findings contradict the view of preschool depression as a transient condition, instead highlighting its role as a predictor of later depressive disorders. In light of this evidence, the present study aims to further investigate the psychopathological trajectories of preschool depression, with a particular focus on its continuity, specificity, and long-term clinical implications. Understanding the developmental course of preschool depression is essential for early identification, timely intervention, and the development of preventive strategies to mitigate the risk of chronic affective disorders later in life.

## Clinical presentation and diagnostic challenges of preschool-onset depression

Preschool depression refers to a clinically significant presentation of major depressive disorder (MDD) occurring in children aged approximately 3 to 6 years characterized by sadness, irritability, anhedonia, excessive guilt, disturbed sleep and appetite, and decreased activity levels (4). In preschoolers, depressive symptoms can present in less overt or disruptive forms, making early recognition by caregivers and clinicians particularly challenging (19). The clinical relevance of preschool depression is further supported by the inclusion of specific diagnostic criteria for early childhood depression in authoritative classification systems, such as the DC:0–5 Diagnostic Classification of Mental Health and Developmental Disorders of Infancy and Early Childhood (20) and the framework proposed by Egger et al. (21). The diagnostic criteria are summarized in **Table 1**.

**Table 1:** Preschool depression diagnostic criteria (20, 21).

Criteria	Description
A.	Depressed or irritable mood across all activities, present most days for at least two weeks, as indicated either by the child’s direct verbalization (e.g., “I’m sad”) or by the observations of others (e.g., the child appears sad or tearful, displays a low mood, or has frequent temper outbursts).
B.	Anhedonia – marked loss of interest or pleasure in all, or almost all, activities (e.g., initiating play or interacting with the caregiver), present most days for at least two weeks, as inferred from the child’s verbal expressions or observed by others. In children, anhedonia may manifest as decreased engagement, responsiveness, and reciprocity.
C.	Two or more of the following must also be present: A significant change in appetite or failure to gain weight consistent with expected developmental growth curves; Insomnia (e.g., difficulty falling or staying asleep) or hypersomnia, present most days for at least two weeks; Psychomotor agitation or retardation observable by others during activities, present most days for at least two weeks; Fatigue or loss of energy, which may appear as reduced enthusiasm during activities, present most days for at least two weeks; Feelings of worthlessness, excessive guilt, or self-blame, evident in play or through the child’s direct verbalization, present most days for at least two weeks; Diminished ability to concentrate or make choices during activities, present most days for at least two weeks; Recurrent references to death or suicide, or self-harming behaviors, as expressed verbally, through play, or through behavior.
D.	The child’s symptoms, or the caregiver’s adaptations to these symptoms, must significantly impair the functioning of the child and/or family in one or more of the following domains: causing distress to the child, interfering with the child’s relationships, limiting the child’s participation in developmentally appropriate activities or routines, restricting the family’s participation in daily routines, hindering the child’s ability to learn and acquire new skills, or interfering with developmental progress.
Age	Diagnosis should be made with caution in children under 24 months of age.
Duration	Symptoms must be present on most days for a minimum duration of two weeks.

Although the clinical presentation of preschool depression may differ from that observed in older children, it is associated with significant functional impairment and increased developmental risk. In a 2010 review, Luby and colleagues (19) highlighted that preschool depression is not a passing phase, but a chronic and recurrent condition. It is characterized by familial aggregation, global impairment in daily functioning, emotional dysregulation, and longitudinal continuity, meaning that symptoms often persist or re-emerge over time. Even when symptoms appear subtle, the disorder can have a substantial and lasting impact on the child’s emotional, cognitive, and social development.

One interesting study by Luby et al. (4) found that depressed preschoolers more frequently exhibited typical affective symptoms, such as loss of interest, guilt, and changes in sleep or appetite, rather than “masked” symptoms like somatic complaints or oppositional behavior. In fact, all DSM-IV symptoms of MDD occurred significantly more often in the depressed group compared with both the psychiatric (p <.001) and healthy control groups (p <.001), including sleep disturbances (p <.01) and concentration problems (p <.01). Furthermore, boys exhibited more violent or destructive play themes than girls (p <.005). In addition, the depressed group consistently scoring higher than both comparison groups in depression/anxiety (p <.001), withdrawal (p <.001), and somatization (p <.001). Overall, sadness and irritability emerged as the most sensitive indicators of depression in early childhood, whereas anhedonia appeared to be the most specific marker distinguishing depressed preschoolers from their peers.

To further examine the clinical presentation of preschool depression, Luby et al. (22) investigated the existence of a melancholic subtype by focusing on the role of anhedonia. In fact, they found that children with anhedonia exhibited a more severe clinical profile characterized by psychomotor slowing (p <.05), decreased reactivity to pleasurable stimuli (p <.01), heightened cortisol reactivity to stress (p <.05), and a stronger familial history of depression (p <.01). Moreover, anhedonic depressed preschoolers were significantly more likely than both healthy (p = .001) and psychiatric comparison groups (p <.01) to display emotional unreactivity to positive events. These findings support the presence of a distinct melancholic subtype of depression emerging as early as the preschool years.

In addition, Luby et al. (7) also investigated the role of self-conscious emotions, specifically shame and maladaptive guilt, in preschoolers. The results showed that depression was significantly associated with higher levels of shame (p < 0.05). Moreover, depressed children exhibited significantly higher levels of guilt compared to their non-depressed peers (p <.001). These maladaptive emotional traits appear to emerge early in development and may evolve into enduring affective features associated with depression in adulthood.

Also neurobiological evidence strongly supports the clinical relevance and developmental impact of preschool-onset depression. In a longitudinal neuroimaging study, Luby et al. (23) demonstrated that preschool-onset depression was significantly associated with accelerated reductions in both cortical gray matter volume (p = .02) and cortical thickness (p = .003). Notably, these neuroanatomical changes were found to be bilateral and widespread, affecting the brain globally rather than being localized to specific regions. Moreover, the observed structural alterations occurred independently of familial risk factors or exposure to traumatic events, highlighting the unique impact of early depressive symptoms themselves on neural maturation. These findings underscore the importance of recognizing preschool-onset depression as a biologically significant condition, with the potential to alter critical neurodevelopmental trajectories during a period of heightened brain plasticity.

Complementing this, cross-sectional data in preschoolers with several levels of depressive symptoms show reduced global cortical surface area (p = .005) and smaller lateral orbitofrontal surface area, a key reward-related region, (p = .0001), with no association with cortical thickness (23). These findings highlight the importance of recognizing preschool depression also as a biologically significant condition, with the potential to alter critical neurodevelopmental trajectories during a period of heightened brain plasticity.

Taken together, this body of research outlines a clear picture of preschool depression as a valid and clinically relevant syndrome, with distinct symptomatology and emotional features, and long-term developmental consequences. Core markers include anhedonia, excessive guilt, psychomotor changes, emotional unresponsiveness, shame, and impaired reparative behaviors—indicators that may signal a more severe melancholic profile. These emotional and biological traits emerge early and may remain stable, increasing long-term vulnerability, underscoring the need for early detection and tailored intervention strategies suited to this developmental stage.

## Understanding risk factors in preschool depression: implications for early identification and prevention

While the clinical characterization of preschool depression has become increasingly refined, the risk factors predicting its onset remain only partially understood. A meta-analysis by Madigan et al. (24), encompassing 71 studies, examined the impact of maternal prenatal stress, specifically anxiety and depression, on children’s socioemotional development, defined as the capacity to regulate and express emotions appropriately. The findings revealed that prenatal stress significantly compromised socioemotional outcomes (p <.001), with a stronger effect for maternal depression than anxiety (p = .009). Notably, prenatal depression increased the risk of socioemotional difficulties by 76%, and prenatal anxiety by 47%, indicating a substantial influence of maternal mood disturbances during gestation on child emotional development.

While Madigan et al. (24) highlighted the impact of maternal stress during pregnancy on children’s later socioemotional development, subsequent studies have focused on the postnatal period, examining how early environmental and familial factors shape the emergence of depressive symptoms. Luby et al. (25) found that a family history of mood disorders was significantly associated with greater depression severity over time in preschoolers. Importantly, children with this familial risk were also more likely to have experienced stressful life events early in life, and early stress played a mediating role between genetic vulnerability and depression severity. These findings highlight the interplay between inherited risk and early environmental adversity, supporting the need for early identification of children at heightened risk.

Extending these results, Bufferd et al. (26) found that early life stress predicted depressive symptoms (p = .05), particularly through its interaction with temperamental fear and inhibition. Interestingly, these stressors were predictive primarily in children without a parental history of mood disorders, suggesting different developmental trajectories. Additional predictors included low inhibitory control (p <.01), parental anxiety disorders (p <.05), and exposure to early adversity (p <.001). These results point to multiple risk pathways for preschool depression: one involving familial psychopathology, and another regards the interaction between temperament and environmental stressors.

More recently, Hopkins et al. (27) adopted a biopsychosocial framework to examine a range of risk and protective factors, including socioeconomic status, parenting stress, parental depression and hostility, child negative affect, effortful control, and attachment security. They found that parental depression at age 5 strongly predicted depressive symptoms in children at age 6 (p <.001). Conversely, higher levels of regulatory temperament traits such as effortful control and self-regulation at age 5 were associated with reduced depression severity one year later (p <.001), suggesting that these regulatory abilities may serve as protective factors, and that enhancing them could help prevent the progression of depressive symptoms. Additionally, secure attachment at age 4 predicted higher EC at age 5 (p <.001), which in turn was associated with fewer depressive symptoms at age 6 (p <.001), highlighting the developmental interplay between attachment and self-regulation.

Consistent with this evidence, Martucci et al. (28)further examined the association between parental psychopathology and preschool depression, focusing on how parental mental health influences children’s clinical outcomes. In their study, preschoolers diagnosed with MDD with both parents affected by psychopathology exhibited greater symptom severity and poorer treatment response (p = .007). Moreover, improvements were observed primarily among children showing externalizing or emotionally dysregulated profiles (p = .00), while more inhibited children benefited less. These findings underscore the central role of parental mental health in shaping both the onset and course of depressive symptoms, emphasizing the importance of considering family-level risk when developing early intervention strategies.

In summary, current evidence suggests that psychological, and environmental factors, such as parental psychopathology, child temperament, early stress exposure, and the development of regulatory capacities, interact to shape distinct developmental pathways toward preschool-onset depression. These findings emphasize the importance of early, multidimensional prevention strategies targeting modifiable risk and protective factors during this sensitive developmental window.

## Early-onset, long-term impact: the developmental trajectory of preschool-onset depression

After establishing the clinical features and early risk factors of preschool depression, it becomes crucial to consider how the disorder develops across subsequent stages of childhood and adolescence. Longitudinal research, as those discussed in this paragraph, has increasingly demonstrated that depression emerging in the preschool years follows a distinct developmental trajectory. Rather than being a transient or developmentally limited condition, early-onset depression often follows a stable and enduring course that extends into middle childhood, adolescence, and even beyond, with significant consequences for emotional, behavioral, and functional development. Longitudinal research has consistently shown that depression beginning in the preschool years is not a transient phase but a clinically significant and developmentally persistent condition. In a longitudinal study, Luby et al. (8)followed 246 children from ages 3–5.11 and found that preschool-onset depression was the strongest independent predictor of full DSM-5 MDD during school age (p = .002). More than half (51.4%) of the children with early-onset depression went on to develop MDD by school age, compared to just 23.8% of those without a history of early depression. This association remained significant even when accounting for other potentially influential factors such as maternal depression (p=.258), family income (p=.223), exposure to trauma (p=.444), and unsupportive parenting (p=.017). Moreover, preschool depression also predicted the later emergence of anxiety disorders (p<.00001) and Attention-Deficit/Hyperactivity Disorder (ADHD) (p= .0002), suggesting both homotypic continuity, with depression itself, and heterotypic continuity, toward other forms of psychopathology.

Consistent with these findings, Silver et al. (29) followed children from preschool to age 15 and found an earlier onset of recurrent depressive episodes among those with preschool-onset depression (p=.05) and a higher risk of recurrence over time (p=.04); they also reported higher odds of later anxiety disorders (p=.045). These results provide additional longitudinal evidence of both homotypic and heterotypic continuity across development.

Complementing these findings, Luby et al. (7) demonstrated that depressed preschoolers at baseline were significantly more likely to meet criteria for MDD again at 12- and/or 24-month follow-up, compared both to children without psychiatric disorders (p <.001) and to those with other psychiatric diagnoses (p <.001). Specifically, 57% experienced at least one additional depressive episode, and 19% met criteria for chronic depression. Even recovered children continued to manifest elevated residual symptoms, highlighting the persistent nature of early-onset depression. These results highlight the homotypic continuity of early-onset depression and its similarity in course to MDD observed in older children and adolescents.

Expanding upon previous findings, Gaffrey et al. (30) investigated whether depression emerging in the preschool years continues to predict the presence of MDD into adolescence and beyond puberty, a developmental stage associated with a significant increase in depression rates. Drawing on data from 270 participants, the study found that preschool-onset depression significantly predicted both early (p <.0001) and late pubertal depression (p = .021), meaning that children with a history of preschool depression were two to three times more likely to experience depression in adolescence compared to those without such a history. Notably, preschool depression was the only early diagnosis that retained significant predictive power for adolescent MDD (p = .0296), underscoring its unique prognostic value. Regarding symptom trajectory, children with a history of preschool depression exhibited a ‘U-shaped’ pattern: depressive symptoms tended to decrease in late childhood but rose again during adolescence. This trajectory suggests the typical developmental course but remained consistently elevated in comparison to peers without early depression. These findings suggest that early-onset depression does not necessarily alter the shape of symptom change over time but rather raises the baseline level of risk across development. In addition, preschool depression predicted a variety of heterotypic outcomes in adolescence (as previously reported by Luby et al. (8),), including ADHD, conduct disorder, and oppositional defiant disorder, further supporting its broader influence on later psychopathology.

Extending this trajectory to clinically significant outcomes, Hennefield et al. (31) found that preschool-onset depression was associated with a markedly increased risk of suicidal thoughts and behaviors (STBs) by preadolescence, with affected children being 7.38 times more likely to report any STBs (p <.001), 6.71 times more likely to have attempted or engaged in suicidal behavior (p = .012), and 8.98 times more likely to endorse STBs in the past month (p = .005) compared to nondepressed peers. Among children with preschool-onset depression, those exhibiting STBs in preschool were 3.46 times more likely to report them again at preadolescence (p = .018), indicating substantial continuity of suicidal risk across development.

Findings from Lijster et al. (32) support these conclusions from a large-scale cohort study involving 7,499 children identifying four distinct depressive symptom trajectories: low, decreasing, increasing symptoms up to age 6 followed by a decline, and increasing without symptoms reduction. Children in the increasing and preschool-limited trajectories showed rising symptoms to borderline clinical levels by age 10, and were more likely to experience poorer psychosocial functioning, including lower self-esteem (p < 0.001) and friendship quality (p 0.006), as well as school-related difficulties (p < 0.001) such as academic underperformance (p 0.060). These findings reinforce the notion that early-emerging depression symptoms, even if subclinical, are associated with meaningful impairments later in childhood.

Taken together, the findings reviewed highlight that preschool-onset depression is not a transient emotional state, but the starting point of a potentially enduring psychopathological trajectory. This trajectory is characterized by elevated symptom severity, chronicity, and recurrence, as well as by both homotypic continuity, reflected in the persistence or re-emergence of depressive symptoms, and heterotypic continuity, with increased risk for other disorders such as anxiety, ADHD and conduct disorder. Importantly, these outcomes are observed even in children with subclinical early symptoms, and are often accompanied by functional impairments in academic, social, and emotional domains. Recognizing and addressing the early trajectory of depressive symptoms is therefore critical to preventing the consolidation of more severe and widespread forms of psychopathology later in development.

## Treatment approaches for preschool depression: evidence-based interventions and innovations

The evidence highlights the critical importance of early identification and intervention for depressive symptoms emerging in preschool-aged children, given their potential for persistence and impact on later development. Depression in early childhood represents a clinically significant condition that is often underestimated, despite its association with long-term emotional, cognitive, and relational difficulties. Timely intervention can interrupt maladaptive trajectories and promote adaptive developmental pathways.

A major innovation in the treatment of preschool depression is the Parent-Child Interaction Therapy – Emotion Development (PCIT-ED), a dyadic psychotherapy derived from the traditional PCIT model originally designed for disruptive behaviors. PCIT-ED integrates emotion-focused components to address the developmental vulnerabilities in emotion understanding and regulation that characterize early-onset depression (33). The treatment emphasizes enhancing parental sensitivity, validation of the child’s affect, and the creation of a secure emotional climate, thereby fostering both symptom reduction and socio-emotional competence (33–35).

PCIT-ED consists of three modules, Child-Directed Interaction (CDI), Parent-Directed Interaction (PDI), and Emotion Development (ED), delivered over 14–20 sessions (see **Table 2**). CDI strengthens positive parent–child exchanges through play; PDI improves limit-setting and compliance; ED explicitly targets emotional awareness and regulation by coaching parents to recognize and respond empathically to their child’s emotions. The therapist provides *in-vivo* guidance through a headset, allowing real-time feedback during parent–child interactions (35, 36). In addition, Lenze et al. (34) showed that PCIT-ED produced large reductions in depressive severity (p<.01), functional impairment (p<.01), and internalizing symptoms (p<.01), with five of seven children no longer meeting diagnostic criteria for depression after treatment. A subsequent pilot randomized trial (36) confirmed these findings, demonstrating greater gains in emotion recognition (p=.002) and executive functioning (p=.011) among children receiving PCIT-ED compared to a psychoeducational control, alongside significant within-group reductions in depression (p<.001), emotional liability (p<.01), and parenting stress (p<.01).

**Table 2:** Parent-child interaction therapy - emotion development.

Phase	Description	Main objectives	Approximate duration
Child-Directed Interaction (CDI)	The parent follows and supports the child’s play, reinforcing positive behaviors and promoting trust.	Strengthen the parent-child relationship through play	3 sessions
Parent-Directed Interaction (PDI)	The parent learns to give clear, consistent commands and manage noncompliance and problematic behaviors.	Reduce disruptive behaviors and improve discipline	3 sessions
Emotion Development (ED)	The parent is guided to recognize, accept, and respond to the child’s emotions, fostering emotional regulation.	Promote emotional regulation and parental empathy	8 sessions
*In Vivo* Coaching	The therapist provides immediate feedback to the parent during interactions with the child via a “bug in the ear.”	Apply strategies in real time	Throughout all sessions

In addition, another study by Luby et al. (35) established PCIT-ED as the first empirically supported psychotherapy for preschool-onset depression. In a sample of 229 parent–child dyads, children who received PCIT-ED were significantly less likely to meet diagnostic criteria for MDD at post-treatment (p <.0001) and showed marked improvements in depression severity (p <.0001), global functioning (p <.0001), and emotion regulation (p <.0001). Parents also reported significant reductions in depressive symptoms and parenting stress (p <.05), confirming both the efficacy and feasibility of the model.

A more detailed, module-specific analysis by Luby et al. (37) revealed that depressive symptoms declined during both the Child-Directed Interaction (CDI) and Emotion Development (ED) phases (PFC-Scale p <.001 for both). However, the ED module uniquely improved CBCL depression scores (p <.001) and parental stress (p <.001). Moreover, ERP data indicated increased neural reward responsiveness following the ED module (p = .007), suggesting that the emotion-focused sessions directly influence affective neurodevelopmental mechanisms.

The maintenance of treatment gains was confirmed of these treatment effects was further demonstrated in the longitudinal follow-up study by Luby et al. (38). Twelve months after treatment completion, children who had received PCIT-ED were significantly less likely to experience a recurrence of depression (p <.001) and continued to show superior global functioning (p <.001) and emotion regulation (p <.01) compared with controls.

Expanding on these findings, Whalen et al. (39) examined whether PCIT-ED produced measurable changes in parenting. Results showed significant reductions in dismissive (p <.001), disapproving (p <.001), and authoritarian (p <.001) styles, together with increases in emotion coaching (p = .04), authoritative parenting (p = .01), and positive affect (p = .001). Observed interactions also revealed a higher positive-to-negative affect ratio (p <.001) and a more positive interactional style (p = .001). These results indicate that PCIT-ED enhances emotional responsiveness and synchrony within the parent–child relationship.

Finally, Donohue et al. (40) identified children’s maternal representations as moderators of treatment response. The effects of PCIT-ED on diagnostic remission were stronger for children with fewer negative maternal representations (p = .01) and for those whose positive representations outweighed negative ones (p = .02). Observed parenting behaviors, however, did not predict outcomes, suggesting that children’s internal representations of the caregiver may influence the extent to which they benefit from treatment.

Together, these studies position PCIT-ED as a developmentally sensitive, mechanism-focused, and family-centered treatment capable of altering both behavioral and neurobiological pathways of early-onset depression. The integration of emotion coaching and parent–child synchrony appears central to its sustained effects on child well-being and relational functioning.

## Conclusion

Preschool depression is a valid and clinically significant condition that deeply impacts a child’s emotional, cognitive, and social development. Far from being a transient developmental phase, it follows a chronic and recurrent course, with both homotypic and heterotypic continuity into later childhood, adolescence, and adulthood.

Core symptoms such as anhedonia, excessive guilt, and emotional dysregulation, along with neurodevelopmental correlates including cortical thinning and reduced gray matter volume, highlight the disorder’s early differentiation and clinical significance.

Current evidence identified several risk factors including genetic and temperamental vulnerability, early life stress and parental psychopathology. However, protective factors such as effortful control, secure attachment, and emotionally responsive caregiving. highlights the potential for preventive interventions targeting early regulatory processes.

In this context, early identification and timely intervention are crucial. Innovative therapeutic approaches like Parent-Child Interaction Therapy – Emotion Development offer developmentally tailored frameworks for intervention that not only alleviate symptomatology but also promote foundational emotional competencies and relational security. Early detection and intervention during this sensitive neurodevelopmental period are therefore critical, both to mitigate immediate impairment, to reduce symptom severity and to alter maladaptive trajectories that may otherwise consolidate into lifelong psychopathology.
